# The shape of a defense-growth trade-off governs seasonal trait dynamics in natural phytoplankton

**DOI:** 10.1038/s41396-020-0619-1

**Published:** 2020-03-03

**Authors:** Elias Ehrlich, Nadja J. Kath, Ursula Gaedke

**Affiliations:** Department of Ecology and Ecosystem Modelling, University of Potsdam, Am Neuen Palais 10, 14469 Potsdam, Germany

**Keywords:** Food webs, Freshwater ecology, Theoretical ecology

## Abstract

Theory predicts that trade-offs, quantifying costs of functional trait adjustments, crucially affect community trait adaptation to altered environmental conditions, but empirical verification is scarce. We evaluated trait dynamics (antipredator defense, maximum growth rate, and phosphate affinity) of a lake phytoplankton community in a seasonally changing environment, using literature trait data and 21 years of species-resolved high-frequency biomass measurements. The trait data indicated a concave defense-growth trade-off, promoting fast-growing species with intermediate defense. With seasonally increasing grazing pressure, the community shifted toward higher defense levels at the cost of lower growth rates along the trade-off curve, while phosphate affinity explained some deviations from it. We discuss how low fitness differences of species, inferred from model simulations, in concert with stabilizing mechanisms, e.g., arising from further trait dimensions, may lead to the observed phytoplankton diversity. In conclusion, quantifying trade-offs is key for predictions of community trait adaptation and biodiversity under environmental change.

## Introduction

Identifying trade-offs between functional traits of species is central to ecology because it provides a fundamental basis to understand species coexistence and the trait composition of natural communities [1]. Trade-offs emerge through physiological, energetic, behavioral, genetic or resource allocation constraints [2] and can be detected within one species [3, 4] as well as on the community level among different species sharing similar individual-level constraints [5, 6]. Such interspecific trade-offs promote species diversity and guide the way of community trait changes under altered environmental conditions [7, 8].

**Fig. 1:**
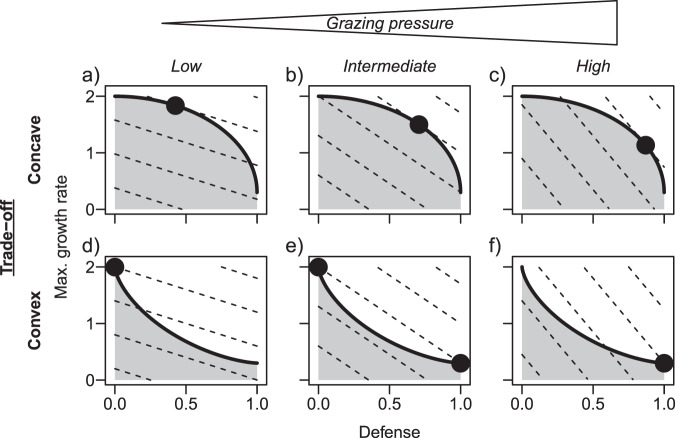
The shape of the trade-off curve in concert with the environment determines the strategies of maximal fitness in a community. Our example considers a trade-off between defense and maximum growth rate (d^−1^) in a prey community with grazing pressure as a biotic environmental factor. The trade-off curve (thick solid line) represents the boundary of the set of feasible trait combinations (gray area) and may be, for example, (**a**–**c**) concave or (**d**–**f**) convex. The fitness landscape is shaped by the grazing pressure (low, intermediate or high), resulting in different slopes of the fitness isoclines (dashed lines). The trait combinations reaching the highest fitness isocline are fitness maxima (dots) and are positively selected. If two or more trait combinations are of maximal fitness in the long term, the respective species with these trait combinations coexist (**e**), otherwise only one species survives (**a**–**d**, **f**).

Theory indicates that it is the shape of the trade-off curve between two traits, reflecting costs of trait adjustments, that determines species coexistence and how trait values change in response to environmental forcing [9–11]. We summarize the theory and specify predictions in Box 1 and Fig. 1. While theory revealing the importance of the shape of the trade-off curve for coexistence and trait dynamics is well developed [12–14], its empirical verification has been left far behind. Two studies successfully tested the theory in small-scale lab experiments assembling different bacterial strains [15, 16]. However, respective approaches from the field are lacking, leaving open the question how the trade-off shape affects the trait composition of natural communities. In this article, we combine theory and long-term field data to provide evidence for the frequently postulated defense-growth trade-off and to show how its shape affects seasonal trait dynamics of phytoplankton in a large European lake.

Phytoplankton communities are well-suited for addressing this issue as important functional traits of phytoplankton have been measured in the lab revealing key trade-offs [17, 18]. Phytoplankton communities are extremely diverse spanning a large trait space [19, 20] indicating that trade-offs play a decisive role in maintaining their biodiversity, although the number of limiting factors allowing for niche differentiation seems to be low compared with the high number of coexisting species (known as Hutchinson’s “Paradox of the Plankton” [21]). Furthermore, phytoplankton species have short generation times allowing for pronounced seasonal succession [22]. This offers the opportunity to observe species sorting in response to recurrently changing environmental conditions driving community trait dynamics.

Previous trait-based studies on phytoplankton communities already quantified trade-offs among different resource utilization traits [5] and revealed how the trait composition of phytoplankton communities in different lakes and a marine system depended on light and nutrient conditions [23–26]. However, phytoplankton can also be strongly affected by herbivory selecting for phytoplankton defense, which was not considered in these studies but is likely to have a crucial effect on their seasonal trait dynamics [22]. Defense against predation often comes at a physiological cost [18], like a lower maximum growth rate [27, 28] or a lower competitive ability [3]. Competitive ability is used here in the sense of Tilman: [6] a high competitive ability refers to a low equilibrium resource concentration in monoculture (R*), where growth equals mortality. Hence, the competitive ability of a species is defined by its resource uptake affinity, but may also depend on its maximum growth rate, especially at high rates of background mortality. Trade-offs between defense and competitive ability or maximum growth rate can mediate antagonistic effects of top-down and bottom-up control on the trait composition. A large body of theory assumes such trade-offs between defense and maximum growth rate [29, 30] or between defense and competitive ability [31, 32]. However, there is no study that empirically quantifies the shape of these trade-offs and uses this information in combination with theoretical insights on trade-off curves (see Box 1 and Fig. 1) to explain how predation and abiotic conditions drive the trait dynamics and variation of natural communities.

**Fig. 2:**
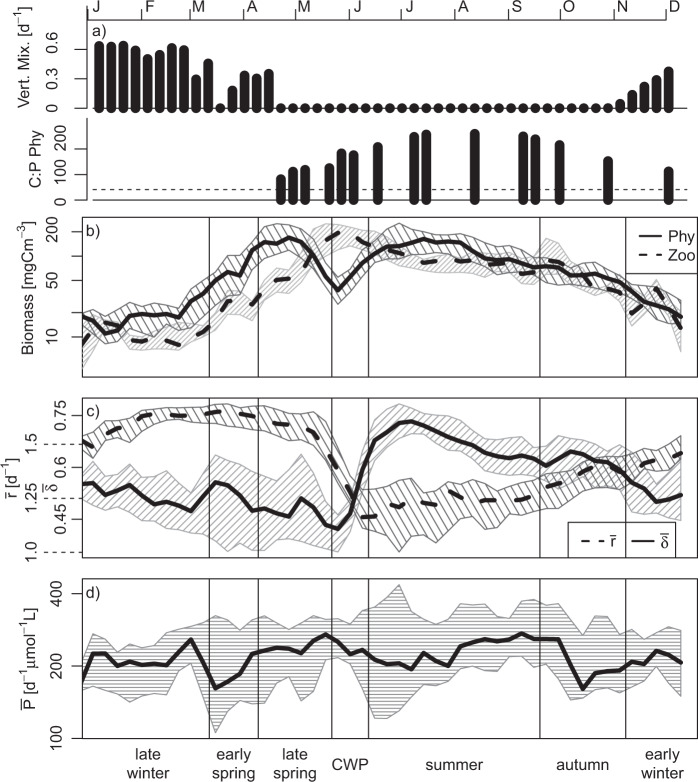
Seasonal dynamics of abiotic factors, phytoplankton and zooplankton biomasses, and phytoplankton community average trait values in a standardized year. **a** The vertical mixing intensity (Vert. Mix.) quantifies the relative amount of phytoplankton exported from the euphotic zone (0–20 m) to larger depths (20–100 m). The carbon to phosphorous (mass) ratio of phytoplankton, C:P Phy, indicates the degree of nutrient depletion (dashed line marks the Redfield ratio), **b** total biomasses of phytoplankton (Phy) and herbivorous zooplankton comprising ciliates, rotifers and herbivorous crustaceans (Zoo), and (**c**, **d**) phytoplankton community average trait values (maximum growth rate $\bar r$, defense $\bar \delta$, and phosphate affinity $\bar P$). **b**–**d** Interannual medians (lines) and interquartile ranges (shaded areas) are shown for the biomasses and the community average trait values. CWP denotes the clear-water phase. For methodical details see the methods and Appendix 1.

Here, we use 21 years of high-frequency density measurements of a natural lake phytoplankton community (large, deep, mesotrophic Lake Constance) and literature trait data (defense against predation, maximum growth rate, and phosphate affinity) to identify how potential interspecific trade-offs govern the community trait dynamics under seasonally changing environmental conditions. In the studied lake, zooplankton grazing, vertical mixing, and phosphate depletion are important limiting factors of phytoplankton [33, 34], which all undergo a highly repetitive seasonal succession (Fig. 2a, b). As for the environmental factors, we found distinct seasonal dynamics in the community average values of defense and maximum growth rate, but not for phosphate affinity (Fig. 2c, d). The phytoplankton trait values were taken from Bruggeman, who obtained the trait values from a statistical model fed with lab trait measurements, phylogenetic and allometric relationships [35]. From this data set, we could infer a distinct concave trade-off between defense and maximum growth rate for the Lake Constance phytoplankton community. Phosphate affinity showed no strong relationships to these traits, but we found slight evidence for a multidimensional trade-off. We parameterized a phytoplankton model with the observed concave defense-growth trade-off, which reproduces the seasonal shift in the biomass-trait distribution within the trait space. For comparison, we parameterized the model also with a hypothetical convex trade-off, which fails in reproducing the observed pattern. This reveals the importance of knowing the exact trade-off shape for understanding trait dynamics. Furthermore, in reference to modern coexistence theory [36], we discuss how low fitness differences of species found along the trade-off curve can promote the maintenance of the large diversity in this community, when taking into account stabilizing mechanisms arising from further trait dimensions and environmental fluctuations.

Box 1 Theory on trade-off curves, fitness landscapes, survival of species and trait dynamics The survival of species and the trait dynamics within a community depend on the species trade-offs between functional traits, quantifying the costs of trait adjustments, and the environmental conditions that determine the fitness landscape. The *trade-off curve* is defined as the boundary of the set of feasible trait combinations, representing all possible phenotypes of species (Fig. 1) [60]. The trade-off curve is fixed by individual-level constraints (e.g., energetic or physiological constraints) and may have different shapes (e.g., concave or convex, Fig. 1), reflecting different costs of trait adjustments.

Species fitness is defined as the net per capita growth rate [61]. The fitness landscape within a two-dimensional trait space can be represented by *fitness isoclines* (Fig. 1) [62], connecting trait combinations of equal fitness. The slope of these fitness isoclines depends on the abiotic and biotic environmental conditions (e.g., grazing pressure, Fig. 1). Trait combinations along the trade-off curve reaching the highest fitness value represent fitness maxima (Fig. 1). Species with these trait combinations are positively selected and survive in the long term. Species itself can change the fitness landscape in a way favorable for species with other strategies/niches (a stabilizing mechanism), e.g., high densities of fast-growing, undefended species lead to increased predator abundance favouring defended species (and vice versa). In the sense of Chesson’s coexistence theory [36], such stabilizing mechanisms may level out fitness differences between species and allow for their stable coexistence.

Given linear fitness isoclines, implying linear trait-fitness relationships [14], theory predicts that concave trade-offs favor species with intermediate trait combinations (Fig. 1a–c), while convex trade-offs promote species with extreme trait combinations (Fig. 1d–f). Under directionally changing environmental conditions, the fitness maximum moves continuously along a concave trade-off curve driving continued sorting of many different species, which results in changes of the community trait composition. For example, an increasing grazing pressure (e.g., due to a lower mortality of grazers) promotes species with higher defense values at the cost of a decreasing maximum growth rate (Fig. 1a–c). In contrast, for convex trade-off curves, fitness is always maximal for only one or two of the extreme trait combinations depending on the environmental conditions (Fig. 1d–f).

## Material and methods

### Study site and sampling

Upper Lake Constance (Bodensee) is a large (472 km²), deep (mean depth = 101 m), warm-monomictic, mesotrophic lake bordered by Germany, Switzerland and Austria. It has a well-mixed epilimnion and a large pelagic zone [37]. Lake Constance underwent reoligotrophication during which the total phosphorous concentration declined fourfold from 1979 to 1996 leading to an annual phytoplankton biomass and production decline by 50 and 25%, respectively [34]. The reoligotrophication did not qualitatively affect the biomass-trait distribution in respect to defense and maximum growth rate (Fig. S5, Appendix 3) and had little impact on phosphate affinity (Figs. 2d and S6). Thus, it is not further considered.

Plankton sampling was conducted weekly during the growing season and approximately fortnightly in winter, culminating in a time series of 853 phytoplankton biomass measurements from 1979 to 1999 (for details see Appendix 1 and https://fred.igb-berlin.de/Lakebase). Phytoplankton counts and cell volume estimates were obtained using Utermöhl [38] inverted microscopy and were converted into biomass based on a specific carbon to volume relationship [39]. Measurements were taken from the uppermost water layer between 0 and 20 m depth, which roughly corresponds to the epilimnion and the euphotic zone. We aggregated almost all species into 36 morphotypes of phytoplankton comprising individual species or higher taxonomic units that are functionally identical or very similar under the functional classification employed here. This guaranteed a consistent resolution of phytoplankton counts across years and neglects species that were very irregularly encountered. The morphotypes constitute a mean value of 92% and a median value of 96% of total phytoplankton biomass on annual average, with particularly high values from spring to summer. Most of the neglected biomass originates from heterotrophs as *Gymnodinium* spp. and *Ochromonas* spp. not belonging to the phytoplankton *sensu strictu*. Zooplankton was sampled with the same frequency as phytoplankton. Data for all major herbivorous zooplankton groups (ciliates, rotifers, cladocerans, and calanoid copepods) were simultaneously available from 1987 to 1996.

### Seasonal patterns

We subdivided the year into seven consecutive phases: late winter, early spring, late spring, clear-water phase (CWP), summer, autumn and early winter. Each phase was characterized by a well-defined combination of abiotic and biotic factors driving the phytoplankton community (Fig. 2): Strong vertical mixing implying a high phytoplankton net export from the euphotic zone (0–20 m) to deep water layers (20–100 m) occurred during winter and partly early spring (Appendix 1). Grazing pressure was most important during the CWP and summer, and declined toward autumn. Nutrient depletion was most relevant in summer and autumn when vertical mixing, supplying nutrients from larger depths, was absent.

### Trait data and trade-offs

All trait values were consistently taken from Bruggeman [35]. He compiled lab-measurements of traits from the literature for numerous taxa and derived from allometric and phylogenetic relationships a statistical model comprising trait values for these and other taxa. For consistency, we used only the values of this model. We defined defense *δ* as 1—edibility. Bruggeman [35] defined edibility as the rate of prey consumption relative to the rate at which the most commonly reported prey, *Rhodomonas minuta*, was consumed by *Daphnia*, which were both dominant prey and grazers in Lake Constance. *Daphnia* dominated the herbivorous crustaceans from the CWP until autumn [40], and its feeding preferences largely overlap with the other herbivorous groups: The highly diverse ciliate and rotifer communities mainly graze on small, undefended algae, but some specialized species consume also larger morphotypes, which were classified as more defended by Bruggeman [41, 42]. The only calanoid copepod, *Eudiaptomus*, shows also large overlap with the prey spectrum of *Daphnia* [43]. Regarding the phytoplankton taxa considered in our study, 93% of the edibility measurements originated from lab cultures of phytoplankton strains from Lake Constance sampled during the first part of our study period [44]. Thus, we consider the edibility values of Bruggeman [35] to be fairly representative for the grazer community in Lake Constance. Phosphate affinity was defined as maximum growth rate divided by the half-saturation coefficient for phosphate, standardized to continuous illumination and a temperature of 20 °C. All morphotypes, their assigned trait data and taxonomy are listed in Table S1. To detect a potential trade-off, we tested the relationship between traits using the Spearman rank correlation coefficients (not biomass weighted).

### Model

**Fig. 3:**
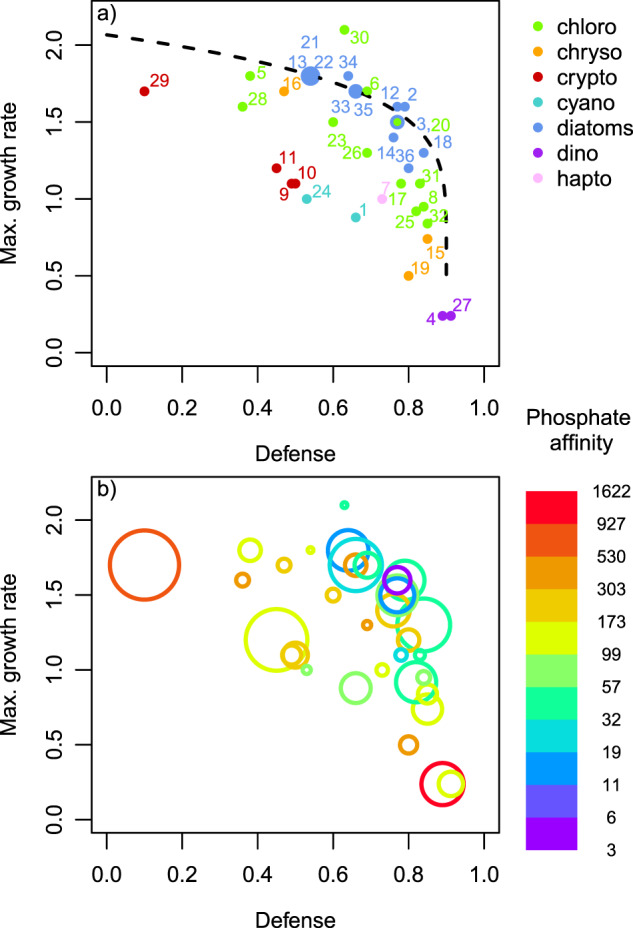
Defense *δ* and maximum growth rate *r* (d^−1^) of the 36 most abundant phytoplankton morphotypes in Lake Constance. **a** Numbers specify the morphotypes (see Table S1 for further details). The dashed line represents the modeled trade-off curve, used for the numerical simulations presented in Fig. 5a, b. Colors indicate different taxonomic groups, i.e., chlorophyta, cryptomonads, chrysophytes, haptophytes, cyanobacteria, diatoms and dinophytes. **b** Colors indicate a third trait dimension, phosphate affinity (d^−1^μmol^−1^L), and the area of the circles is scaled by the mean annual relative biomass of the morphotypes.

We developed a simple food web model to show which phytoplankton trait combinations are favored under low (e.g., during early spring) and high grazing pressure (e.g., during summer). The model included *N* phytoplankton species, which face a defense-growth trade-off, and one zooplankton group:

1 $$\frac{{dP_i}}{{dt}}\, =\, \left( {r_i\frac{R}{{K + R}}\, -\, \frac{{G\left( {1\, -\, \delta _i} \right)Z}}{{H\, +\, \mathop {\sum}\nolimits_{i\, = \,1}^N {P_i} }}\, -\, m_P} \right)P_i,$$

$$\frac{{dZ}}{{dt}}\, =\, \left( {\varepsilon \frac{{G\mathop {\sum}\nolimits_{i\, =\, 1}^N {\left[ {\left( {1\, -\, \delta _i} \right)P_i} \right]} }}{{H\, +\, \mathop {\sum}\nolimits_{i\, =\, 1}^N {P_i} }}\, -\, m_z} \right)Z,$$

where *P*_*i*_ represents the biomass of phytoplankton species *i*, *Z* the zooplankton biomass and *R* the nutrient concentration limiting phytoplankton growth. Assuming a fixed nutrient pool *R*_*max*_, the available nutrient concentration can be written as $R\, =\, R_{max}\, -\, \mathop {\sum}\nolimits_{i\, =\, 1}^N {P_i\, -\, \frac{1}{\varepsilon }Z}$, i.e., the total amount of nutrients minus the nutrients fixed in biomass of phytoplankton and zooplankton [45]. Note that the nutrients are in units of phytoplankton biomass. *r*_*i*_ denotes the maximum growth rate of phytoplankton species *i*, *δ*_*i*_ its defense against zooplankton, *K* the half-saturation constant for nutrient uptake (determined by its nutrient affinity), and *m*_*P*_ the natural mortality of phytoplankton. The latter two are assumed to be equal for all phytoplankton species. *G* represents the maximum grazing rate of zooplankton, *H* the half-saturation constant of zooplankton for phytoplankton ingestion, *ε* the conversion efficiency of phytoplankton biomass into zooplankton biomass and *m*_*z*_ the mortality of zooplankton (for a detailed parameter description see Appendix 4). By changing *m*_*z*_, we vary the importance of grazing pressure on phytoplankton. We run simulations for two different scenarios with constant conditions, i.e., without periodical forcing: (1) *m*_*z*_ is high, and (2) *m*_*z*_ is low, mimicking distinct seasonal phases of low (1) and high grazing pressure (2). From these simulations, we obtain the dominant trait combinations for each phase and the time to extinction of inferior species as a fitness estimate. We assume a concave trade-off curve between *r*_*i*_ and *δ*_*i*_, similar to the one found in the empirical data (Fig. 3), and considered 199 different phytoplankton species with trait values spanning the whole feasible trait space. For details on the justification, parametrization, initialization and numerical integration of the model see Appendix 4.

## Results

The results section is divided into four parts: First, we present insights into seasonal dynamics of abiotic conditions, total phyto- and zooplankton biomasses and phytoplankton community average trait values, defined as the biomass-weighted mean of the trait values of all morphotypes. For these total biomasses and community average trait values, we show the interannual medians and the corresponding interquartile ranges at each standardized sampling date to provide information on interannual variability. Second, we reveal insights on the trade-offs obtained from trait data for the phytoplankton morphotypes encountered in Lake Constance and the mean annual biomass-trait distribution. Third, we show how the biomass-trait distribution changes seasonally in response to altered environmental conditions. Finally, we compare the observed patterns with our model predictions.

### Seasonal dynamics

Abiotic factors and phyto- and zooplankton biomasses showed seasonal patterns typical for a temperate, monomictic lake with winter mixing and phytoplankton spring and summer blooms, the latter under nutrient depletion, and in between the CWP when zooplankton biomass comprising ciliates, rotifers, cladocerans and calanoid copepods was maximal (Fig. 2a, b). Also the phytoplankton community average values of defense $\bar \delta$ and maximum growth rate $\bar r$ exhibited a distinct seasonality (Fig. 2c). $\bar \delta$ was low in late winter and spring with relatively large differences among years. At the end of the CWP, it increased sharply, reached its maximum in summer and declined slowly thereafter. The low interannual variation during this period suggests a high selection pressure on this trait. $\bar r$ exhibited the opposite seasonal trend. It was high in late winter and spring and declined sharply during the CWP with a low interannual variability (Fig. 2c). In summer, $\bar r$ was low and more variable among years and reincreased thereafter. In contrast, the community average phosphate affinity $\bar P$ did not show such a clear and recurrent seasonal pattern (Fig. 2d). The fluctuations of $\bar P$ were small compared with the large trait range (3–1600 d^−1^μmol^−1^L).

### Trade-offs

The 36 dominant morphotypes co-occurring in large, deep Lake Constance covered a large range of values in defense *δ* and maximum growth rate *r* (Fig. 3a). In general, a low value in δ was accompanied by a high value in *r*, and vice versa (Spearman rank correlation coefficient *ρ* = −0.61, *p* = 10^−4^). We found no morphotype that maximizes both *δ* and *r* simultaneously, suggesting a physiological or energetic constraint. Morphotypes with low values in both traits, resulting in low fitness, were not found either, indicating past competitive exclusion. Many morphotypes had an intermediate *δ* and high *r* or vice versa implying a concave trade-off curve. At distinct defense levels, diatoms and chlorophytes had generally higher maximum growth rates than the other morphotypes suggesting that other trait dimensions may play a role as well.

### Mean annual biomass-trait distribution

The mean annual biomass distribution in the *δ*-*r* trait space is obtained by weighting the morphotypes with their relative contribution to the total annual phytoplankton biomass (Fig. 3b). As expected by theory, the biomass was concentrated along the trade-off curve (i.e., for a given value of *δ*, morphotypes with a higher r dominated over those with lower *r*) and at intermediate δ and rather high *r* with some remarkable exceptions. A morphotype at one end of the trade-off curve, exhibiting the lowest *δ*, *Rhodomonas ssp*. (#29, cf. Fig. 3a and Table S1), constituted the highest annual share of biomass of an individual morphotype and occurred in almost every sample although its *r* did not exceed the values of some more defended morphotypes. Among others (for more details, see discussion), its success may be attributable to its very favorable value along a third trait dimension, phosphate affinity (Fig. 3b). We found also substantial biomass at the other end of the *δ*-*r* trade-off curve mostly due to a strongly defended morphotype with a very low growth rate, *Ceratium hirundinella* (#4), which had the highest phosphate affinity of all morphotypes. This pattern can be generalized, as mostly morphotypes with trait combinations further away from the trade-off curve showed higher values in phosphate affinity indicating that fitness losses due to lower *δ* or *r* may be counteracted by higher phosphate affinity (Fig. 3b). For example, a group of diatoms (*Asterionella formosa* (#2), *Fragilaria crotonensis* (#18), *Stephanodiscus neoastreae* (#33), *Stephanodiscus ssp*. (#34)) and the chlorophyte *Cyclotella* ssp. (#12), forming the upper part of the concave *δ*-*r* trade-off curve, grew fast relative to their rather high level of *δ*, but had only low to intermediate phosphate affinity. An exception to that was *Cryptomonas* ssp. (#11) which had intermediate values for all traits but the second highest mean annual relative biomass of all morphotypes (for further trait dimensions, see discussion). Overall, the *δ*-*r* trade-off was much more clearly expressed than the relationship between *P* and *δ* or *r*, respectively (Fig. S2), but we found some indication for a three-way trade-off among *δ*, *r,* and *P*, whereby the trade-off between *δ* and *r* dominated (Online Movie).

### Seasonal dynamics of the biomass-trait distribution

**Fig. 4:**
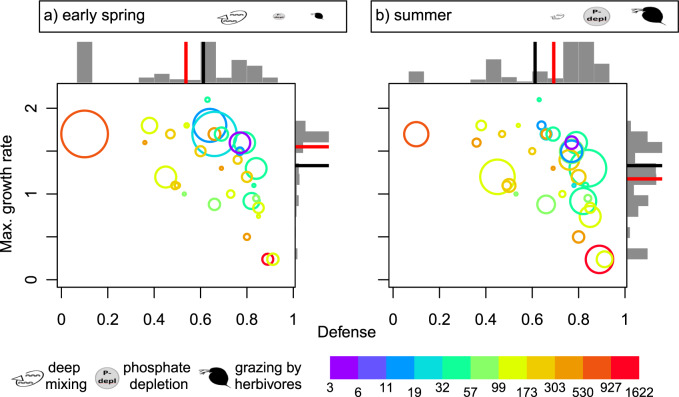
Seasonal differences in the trait space of defense *δ* and maximum growth rate *r* (d^−1^). Positions in the trait space of the 36 most abundant phytoplankton morphotypes in Lake Constance for (**a**) early spring and (**b**) summer. Colors indicate the morphotypes’ phosphate affinity (d^−1^μmol^−1^L) and the area of the circles the mean relative biomasses. The bars display the relative biomass distribution along the two trait axes in each phase. The red lines in the bar plots mark the phase mean of the community average trait values and the black lines display the annual mean of the community average trait values as a reference ($\bar \delta$ = 0.61, $\bar r$ = 1.33). The icons represent the dominant drivers of the phytoplankton community (vertical mixing, phosphate depletion, grazing by herbivores) and their size indicates their relative importance for phytoplankton net growth in each phase.

The biomass distribution within the *δ*–*r* trait space varied systematically during the season (Figs. 4 and S4) in line with pronounced changes of the major forcing factors of phytoplankton development (Fig. 2). For example, in early spring, intensive vertical mixing (resulting in a high export of phytoplankton from the euphotic zone to larger depth) was a dominant driver of the phytoplankton community in deep Lake Constance, while grazing pressure and nutrient depletion were very low (Fig. 2a, b). Accordingly, morphotypes with high *r* being able to compensate for high losses and to exploit the high nutrient concentrations dominated, whereas morphotypes with low *r* and high *δ* were almost absent (Fig. 4a). This is reflected in the seasonal phase means of the community average trait values, $\bar \delta$ = 0.52 and $\bar r$ = 1.57 d^−1^. In contrast during summer stratification, nutrient depletion and grazing pressure were the dominant drivers of phytoplankton (Fig. 2a, b) and the biomass-trait distribution shifted toward morphotypes with intermediate or high *δ* and accordingly lower *r* (Fig. 4b, $\bar \delta$ = 0.69, $\bar r$ = 1.18 d^−1^) (for other seasonal phases see Fig. S4).

### Model results

**Fig. 5:**
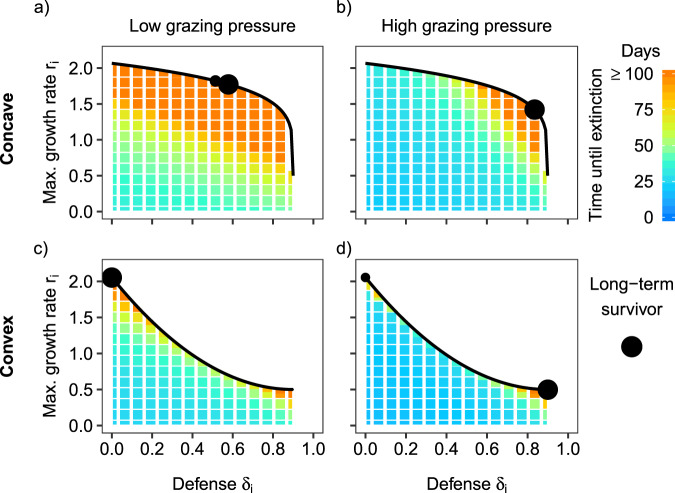
Simulation results for different trade-off curves and grazing pressure. Model predictions for (**a**, **b**) a concave or (**c**, **d**) a convex trade-off curve between defense *δ*_*i*_ and maximum growth rate *r*_*i*_ (black line) in the scenario of low grazing pressure on phytoplankton (*m*_*z*_ = 0.14 d^−1^) mimicking conditions in early spring (**a**, **c**), and the scenario of high grazing pressure (*m*_*z*_ = 0.04 d^−1^) during summer (**b**, **d**). The black dots denote the trait combinations of phytoplankton species which survive in the long term (i.e., the fitness maxima), their size marks the mean relative biomass contribution between day 9000 and 10,000 averaged among 50 simulations with randomized, different initial conditions (see Appendix 4). The color grid displays the average time until extinction of the different trait combinations in the short term, that is, within the first 100 days of the simulations.

A phytoplankton species model, parametrized with the empircally established concave trade-off (Fig. 3a, dashed line), reproduced the general pattern in the data, that is, the favorable trait combinations shift from early spring (low grazing pressure) to summer (high grazing pressure) toward higher *δ*_*i*_ at the cost of a lower *r*_*i*_ (Figs. 4 and 5a, b). For the given concave trade-off curve and set of trait combinations, the model predicted that two very similar species with intermediate *δ*_*i*_ but high *r*_*i*_ coexist in the long-term under low grazing pressure (Fig. 5a). Under high grazing pressure, the long-term outcome of the model was the survival of one species with a high *δ*_*i*_ but intermediate *r*_*i*_ (Fig. 5b, for biomass dynamics see Fig. S7). When considering the short-term results of the model being more in line with the time scale relevant for the data of the different seasons, we found that many species survived along the concave trade-off curve (especially close to the fitness maximum) the first 50–100 days (Fig. 5a, b), in accordance with the observations (Fig. 4). This holds in particular under low grazing pressure (Fig. 5a). Overall, the time until extinction was shorter under high grazing pressure due to the high mortality caused by abundant grazers (Fig. 5a, b). In general, the rate of extinction increased (i.e. fitness decreased) toward the unfavorable edge of the trait space (low *δ*_*i*_, low *r*_*i*_), where the slope of the fitness isoclines depended on the degree of grazing pressure (cf. Figs. 1 and 5a, b). Under high grazing pressure, fitness increased more strongly in the direction of the defense axis than under low grazing pressure (cf. Figs. 1 and see color gradient in Fig. 5a, b).

For a convex trade-off, the model predicted a qualitatively different pattern (Fig. 5c, d). Under low grazing pressure, only the undefended species with the highest *r*_*i*_ survived in the long term (Fig. 5c). Under high grazing pressure, the undefended prey coexisted with the defended species with a very low *r*_*i*_, where the biomass of the defended species exceeded the biomass of the undefended species (Fig. 5d and for biomass dynamics see Fig. S8). In general, the rate of extinction of inferior species was higher compared with the concave case (Fig. 5).

## Discussion

The 36 dominating phytoplankton morphotypes in Lake Constance faced a concave trade-off between defense and maximum growth rate. We found that the community average values of defense and maximum growth rate showed opposed seasonal dynamics. We did not observe distinct seasonal dynamics in the community average phosphate affinity, but morphotypes with a rather low growth rate relative to their defense level often had a relatively high phosphate affinity. Theory predicts that concave trade-off curves promote species with intermediate strategies (Box 1 and Fig. 1). Our data support this prediction as intermediately defended morphotypes with intermediate to high maximum growth rates constituted the largest proportion of total annual phytoplankton biomass. Trait shifts in the phytoplankton community along the concave trade-off curve were exactly in line with seasonal changes of the environmental conditions. The model predicted a shift toward higher defense levels at the cost of lower maximum growth rates with increasing grazing pressure from spring to summer, as found in the data, and revealed low fitness differences of persisting species along the trade-off curve. Assuming a hypothetical convex trade-off in the model, for comparison, produced biomass-trait distributions were qualitatively different from the observations. Hence, our model results highlight the importance of quantitative knowledge on the shape of the trade-off.

The quantification of the trade-off was based on trait data provided by Bruggeman [35]. He obtained phytoplankton trait values from a statistical model, fed with a great quantity of lab trait measurements and phylogenetic relationships. He provided also quantitative information on model uncertainties, i.e., the standard errors of the trait estimates [35]. We consider these uncertainties to be minor compared with the measured trait range, not questioning the general pattern of a concave trade-off (Fig. S1b). The defense (edibility) values, used in Bruggeman’s statistical model [35], were almost entirely based on measurements of Lake Constance phytoplankton strains, sampled during the first part of our study period [44], and were tested for daphnids, the dominant herbivorous crustaceans in Lake Constance, which have a similar food spectrum as most of the other herbivores (see Methods). Hence, we argue that the concave trade-off between defense and maximum growth rate obtained from these trait data is adequate for the considered phytoplankton community in Lake Constance. Wirtz and Eckhardt [46] suggested a linear defense-growth trade-off for the same phytoplankton community, but they considered only seven species, missing several dominant ones. They were able to predict the seasonal dynamics of total phyto- and zooplankton biomass and of the community average trait values [46]. However, they did not consider the biomass distribution in the trait space and thus could not provide predictions on which species/trait combinations may dominate or co-occur. To adequately predict this biomass-trait distribution, the trade-off shape is important. By including trait data on many more than seven species, we found that the trade-off was concave and hence favored species with different intermediate defense levels, as observed.

Bruggeman [35] included also data on cell sizes. We found that defense correlated positively and maximum growth rate negatively with cell size (Fig. S3, Appendix 3), providing a potential mechanistic explanation for the existence of the defense-growth trade-off [47, 48]. However, other cell size-independent defense strategies are relevant as well, e.g., cell wall thickness, colony formation, toxicity and cell shapes, which introduce substantial scatter into the relationship between defense and size.

Our model showed that, for a concave trade-off curve, two very similar species can stably coexist (Figs. 5a and S7). This is in contradiction with theory predicting the survival of only one species (see Box 1 and Fig. 1a–c). The two species have intermediate strategies close to the fitness maximum and coexist based on stabilizing mechanisms arising from their slight difference in defense and growth [36]. However, this community is not evolutionary stable [49]. Given gradual evolution, we expect that one species would reach the exact fitness optimum via trait adaptation and outcompete the others for a concave trade-off curve [10, 12]. Even without evolution, such a coexistence would not last if a species exactly at the fitness maximum is initially present in the community. In line with our model results, Leibold [50] predicted a similar coexistence pattern based on a graphical approach, although his focus was not on the trade-off structure: Two very similar prey species coexisted in a food web with different prey species sharing one resource and one predator and a continuous transition occurred in the set of prey species persisting under gradually changing environmental conditions. With increasing system productivity, species with a higher defense level persisted [50], similar to the pattern in our study when increasing the grazing pressure. This indicates that he implicitly assumed a trade-off structure equivalent to the concave trade-off in our study. We argue that the behavior of communities in respect to coexistence (e.g., coexistence of similar intermediate strategies vs. different extreme strategies) and species replacement under environmental change may allow conclusions on the underlying trade-off structure.

The low trait variation maintained in the long-term model simulations is in contradiction with the empirical data showing a large trait variation, including species having intermediate strategies as well as specialized species (highly defended or fast-growing). The high number of species in such phytoplankton communities, exceeding the number of limiting factors (i.e., potential niches), is well known as the “Paradox of the Plankton” [21]. In the absence of stabilizing mechanisms arising from variation of interacting populations or environments in space or time [36], the number of coexisting species cannot exceed the number of limiting factors [51]. In line with that, our simple model, including only two niche dimensions (i.e., being defended or fast-growing), generated coexistence of maximal two species. Nevertheless, low fitness differences allowed for short-term co-occurrence of species in the model along and slightly below the concave trade-off curve (see green/orange region in Fig. 5a, b), a similar trait space where species persisted in the natural community (Fig. 3b). In contrast, feasible trait combinations well apart from the trade-off curve (low defense and low maximum growth rate) went quickly extinct in the model, implying a high fitness disadvantage. This provides an explanation for their absence in Lake Constance and in the whole data set of Bruggeman [35] (Fig. S1a).

According to modern coexistence theory [36], low fitness differences (as found along the trade-off curve) can form a fundamental basis for long-term maintenance of biodiversity: as then even slight stabilizing mechanisms (i.e., mechanisms slightly increasing negative intraspecific interactions relative to negative interspecific interactions) can lead to stable coexistence of many species. Fitness differences can be very low along a trade-off curve, if its shape is very similar to the shape of fitness isoclines [14, 52], e.g., for a nearly linear trade-off given linear fitness isoclines (Fig. 1). Stabilizing mechanisms, which overcome fitness differences and may help to explain the high trait variation observed along the trade-off curve, can be divided into: (1) niche differentiation along further trait axes, and (2) fluctuation-dependent mechanisms, like relative nonlinearity in competition and the storage effect [36].

Several trait dimensions, not considered in our model, may contribute to the biodiversity in the phytoplankton community, by further reducing fitness differences or by enabling niche differentiation. For instance, a high phosphate affinity is beneficial under strong nutrient depletion during summer and autumn. Although we found no clear increase of this trait on the community level during summer, it may explain the success of certain morphotypes. For example, the defended dinophytes (like *Ceratium hirundinella* and *Peridinium sp*., taxon number #4 and #27 in Fig. 3a) have very high phosphate affinities and constitute substantial biomass, despite their very high defense costs regarding the maximum growth rate (Fig. 3b). The undefended *Rhodomonas* ssp. (#29) also had a high phosphate affinity (Fig. 3a, b), which sheds light on its observed high biomasses and very regular occurrence in spite of its maximum growth rate not exceeding the one of intermediately defended morphotypes. In fact, we found a weak three-dimensional trade-off among defense, growth rate and phosphate affinity (Online Movie), though the negative correlation between the former two was the most striking pattern in the trait data (Fig. S2, Appendix 3).

Different light spectra and phytoplankton photopigmentation represent another important source for niche differentiation [17, 25]. For example, *Rhodomonas* spp. (#29) is able to use additional light spectra, based on the red accessory photopigment phycoerythrin allowing photosynthesis at greater depths, which is relevant year round due to vertical mixing and self-shading. The same holds for *Cryptomonas* spp. (#11) which also reached high biomasses irrespectively of its rather low maximum growth rate relative to its defense level (Fig. 3a, b). The cyanobacteria (*Anabaena* spp. and *Oscillatoria* spp., #1 and #24) also produce additional photopigments, which may compensate for their relative low maximum growth rates (Fig. 3a, b). Motility, in terms of swimming/floating toward light, can increase the performance with respect to light harvesting [17], which is relevant for e.g., the cyanobacteria showing buoyance regulation. Furthermore, vertical migration of some phytoplankton morphotypes, like *Ceratium hirundinella* (#4), enable exploiting additional nutrient sources from deeper water layers, when the water column is stratified during summer. Mixotrophy represents another possibility to obtain additional phosphate, which is relevant in Lake Constance [53]. The low phosphate affinities of bacterivorous mixotrophs as *Dinobryon ssp*. (#15) may partly explain the seasonally and interannually invariant signal in the community average phosphate affinity (Figs. 2d and S6) as they predominantly occur during summer and in later years. Diatoms seem to have maximal fitness regarding their defense and maximum growth rate, and are indeed present at high biomasses (Fig. 3a, b). However, they face disadvantages due to the production of shells, implying an additional silica demand and causing high sedimentation rates during stratified conditions, which leads to lower net growth rates than expected from their maximum growth rate. This helps to explain their success during early spring (Fig. 4a).

Relative nonlinearity in competition and the storage effect represent further stabilizing mechanisms, which may be relevant for our system and both depend on fluctuations in populations or environmental conditions (e.g., nutrient concentrations) [36]. Abrams [11] showed for a competition model that stable coexistence of two specialists using two different resources and one generalist is possible under asynchronous resource fluctuations. The species coexisted based on the relative nonlinearity in their resource uptake functions [51]. Such relative nonlinearity enabling stable coexistence has not been found for the type of predator-prey model considered here, but may be relevant when including additional resources (e.g., silica, light) with seasonally fluctuations. This can lead to coexistence of a high number of phytoplankton species, exceeding the number of limiting resources under nonequilibrium conditions [54, 55]. However, we did not include such seasonal forcing in our model simulations, but run different scenarios with constant environmental conditions mimicking distinct seasonal phases. Based on that, we obtained insights on the fitness landscape, that is, which trait combination would be favored during a certain seasonal phase and which species would be of low fitness (i.e., go quickly extinct in the simulation). The model purpose was not to reproduce the dynamics and the stable coexistence of many species across years in a distinct lake. This would demand a more complex modeling approach implementing, among others, periodical forcing of the abiotic environment (light, vertical mixing intensity, nutrient availability) and details like the overwintering strategies of phytoplankton, which goes beyond the scope of this article.

Lake Constance exhibits a pronounced seasonality (Fig. 2a, b). Our data demonstrate that the instantaneous fitness maximum gradually moves along the trade-off curve from fast-growing, intermediately defended species in early spring to slowly growing but more defended species in summer and then back in winter (Fig. S4, Appendix 3). Thus, different species along the trade-off curve have maximal fitness at different times of the year. This pattern of gradually moving fitness maxima is specific to concave trade-off curves (Fig. 1a–c) and is not expected for convex ones (Fig. 1d–f). Phytoplankton species form resting stages under unfavorable conditions, which buffers population losses [56]. This gives rise to storage effects [36], contributing to the maintenance of numerous phytoplankton species along the trade-off curve.

Lake Constance has successfully served as a model system for large open water bodies including marine ones [57]. It exhibits a typical seasonal plankton succession, driven by vertical mixing, grazing and nutrient limitation [22]. These environmental factors are also main drivers of marine phytoplankton, which is ecologically similar to freshwater phytoplankton and may face similar trade-offs [58]. Trade-offs between defense and growth are also relevant in terrestrial plant communities, for example grasslands [59]. Thus, our findings are likely relevant for numerous ecosystems. Furthermore, our results show that the information on trade-off shapes allows for an understanding of ongoing trait changes directly under field conditions.

Overall, the identification of the major trade-off and its shape provided a remarkable key to understand trait shifts and altering species composition in the phytoplankton community under seasonally changing environmental conditions. Although multiple trait dimensions likely play a role, our results showed that defense and maximum growth rate represent key traits in phytoplankton of Lake Constance, where grazers are known to strongly impact phytoplankton net growth [37]. A high maximum growth rate is beneficial at high resource concentrations, but also at low concentrations, when not coming at substantial costs of a lower nutrient uptake affinity. The maintenance of trait variation was likely promoted by low fitness differences along the concave trade-off curve. Low fitness differences allow coexistence by even slight stabilizing mechanisms arising from niche differentiation along multiple trait axes and fluctuations in environmental conditions, continuously moving favorable trait combinations along the concave trade-off curve. Our study successfully explained major trait dynamics based on a simple model, including only the interspecific defense-growth trade-off, and allowed to verify the theory on trade-off shapes in the field. In conclusion, quantifying trade-off shapes enhances our understanding of trait dynamics and variation in natural communities.

## Supplementary information

Supplemental material of Ehrlich et al. 2020

## Data Availability

The data shown in this paper are available under 10.6084/m9.figshare.11830464.v1.
