# Supplementary material for: The shape of a defense-growth trade-off governs seasonal trait dynamics in natural phytoplankton
Source: ISME J. 2020 Mar 3;14(6):1451–62. doi: 10.1038/s41396-020-0619-1 (PMC7242350; doi:10.1038/s41396-020-0619-1)
Supplement: Supplementary file 1 — Supplemental material of Ehrlich et al. 2020 [file 41396_2020_619_MOESM1_ESM.pdf]

<u>**Supplementary Material**</u>

The shape of a defense-growth trade-off governs seasonal trait dynamics in natural phytoplankton

Elias Ehrlich, Nadja J. Kath and Ursula Gaedke

The supplement includes five appendices. The first appendix contains information about sampling and data processing. The second appendix provides details on the trait data. The third appendix describes supporting results (trait correlations with phosphate affinity and size, and re-oligotrophication patterns). The fourth and fifth appendix include model details and results.

Appendix 1: Methodical details

Classification of seasonal phases

We subdivided the year into 7 consecutive phases to minimize inter-annual variability due to different climatic conditions. These phases are late winter, early spring, late spring, clear water phase, summer, autumn and early winter (Fig. 2). The start and end of each phase was mostly not a fixed calendar date but was determined for each year based upon threshold values of independent physical (vertical mixing, temperature, water transparency), chemical (soluble reactive phosphorous concentration) and/or biological parameters (phytoplankton and zooplankton biomass, chlorophyll concentration and species composition)(1–3). For example, early spring is defined to start when the chlorophyll concentration and algal biomass start to increase, and the clear-water phase when the chlorophyll concentration and algal biovolume fall below a certain value and the Secchi depth surpasses a distinct level.

Each period is associated with a different well-defined forcing regime. (i) First, during late winter deep mixing and low irradiance lead to a decrease of plankton biomass to the annual minimum level. (ii) Early spring is characterized by unstable stratification, variable underwater light climate, low grazing pressure and high, non-limiting nutrient concentrations, which enables the first growth of algae and some grazers interrupted by mixing events. (iii) During late spring algal biomass further increases with the onset of thermal stratification which reduces nutrient concentrations. The high biomass of mostly small, edible algae promotes growth of different groups of micro- and meso-zooplankton. Grazing pressure mostly by ciliates increases. (iv) As a consequence, phytoplankton biomass strongly declines, resulting in the clear water phase, which is characterized by the strongest grazing pressure throughout the year, mainly caused by meso-zooplankton as daphnids. Nutrient concentrations re-increase during the clear water phase due to remineralization and

with decreased grazing pressure, the summer phytoplankton bloom starts. (v) Summer is marked by severe nutrient depletion leading to strong competition within the phytoplankton community, and the relevance of different zooplankton groups (ciliates, rotifers, cladocerans and copepods) with different feeding strategies and grazing on different groups of phytoplankton. (vi) An increase of the mixing depth as autumn begins leads to a minor reduction of algal biomass and replenishing of nutrients from deeper water. The increase in nutrients may give rise to an autumn phytoplankton and crustacean maximum, paralleled by shifts in algal species composition. (vii) Early winter starts in mid of November and is characterized by an increasing intensity of deep mixing and low irradiance.

Standardized time

The duration of the seasonal phases varied among years. To account for this meteorological year-to-year variation, we aligned the sampling dates to a standardized time axis. First, each sampling date (e.g., day 25 in phase 2 in 1986) was scaled relative to the duration of the respective phase in that year (e.g. phase 2 lasts 50 days in 1986) resulting in the relative sampling day (e.g. 25/50). Multiplying the relative sampling day with the inter-annual mean duration of the respective phase (e.g. 46 days) yields the standardized day number of that sampling date (e.g. 25/50*46=23). Based on this method each sampling date can be assigned to a certain week in a standardized year (data see https://doi.org/10.6084/m9.figshare.11830464.v1). To display the seasonal biomass dynamics (shown in Fig. 2), we took the inter-annual median and quartiles of the biomass data for every standardized week and then smoothed the data by averaging the medians/quartiles of two adjacent weeks (moving average).

Aggregation of species into morphotypes

We used an intermediate level of taxonomic resolution distinguishing 36 morphotypes to achieve taxonomic consistency across the long sampling period. Each of these morphotypes contributed at least 5% to the biovolume of total phytoplankton at an individual sampling date during 1979-1982, i.e. the information about rare species got lost for the years 1979-1982. Considering the years 1979-1999, these 36 morphotypes comprise about 92% of total phytoplankton biomass. We omitted counts of morphotypes which could either not be identified, were very rare or encountered only during individual sampling events or short periods. Given its improved reliability for long-term studies we used this dataset in previous studies as well(1,4–6). Details can also be found in LakeBase (https://fred.igb-berlin.de/Lakebase).

Mean relative biomasses

To evaluate the relative importance of a phytoplankton morphotype over the 21 years of sampling, we derived its mean annual relative biomass as follows: First, we calculated the relative biomass of each morphotype for every sampling date. Second, we averaged these relative biomasses among all dates within each year which yields the corresponding annual relative biomass of each morphotype. Finally, we derived the mean of these annual relative biomasses across years. This procedure reduces the influence of outliers at single dates, and gives equal weight to all sampling dates per year and all years, which partly differed in their total biomass and sampling resolution. The relative importance of each morphotype during distinct seasonal phases (e.g., early spring) were computed accordingly by considering only the relative biomasses of the dates within that phase (data see https://doi.org/10.6084/m9.figshare.11830464.v1). The calculated mean relative biomasses allowed to infer the respective biomass-trait distributions since each morphotype represented a specific trait combination.

C:P ratio

We used the cellular carbon to phosphate mass ratio of phytoplankton (C:P) as an indicator for nutrient depletion which was measured at the standard sampling site in 1995 (data see https://doi.org/10.6084/m9.figshare.11830464.v1)(7). The cellular C:P is more informative than the ambient phosphorous concentration in the water as phytoplankton can store phosphorous. Furthermore, most phytoplankton species can take up substantial amounts of phosphorous even at concentrations below the detection limit as they prevail in Lake Constance throughout summer.

Vertical mixing intensity

Given the large depth of the lake, most biological activity and thus sampling effort was concentrated on the upper 0-20 m depth. Thus, we report here the mean biomasses averaged across this water layer. As the surface phytoplankton concentration is usually much higher than in deep strata, deep vertical mixing (i.e. down to 60 or 100m depth) implies a net export from the surface to larger depths. To quantify this phytoplankton export, the vertical mixing intensity was inferred from a one-dimensional hydrodynamic K - ε turbulent exchange model(8,9) and expressed as net exchange rate from the uppermost layer (0-8 m) to the deepest layer (20-100 m). Its temporal dynamics is closely related to the net exchange rate from 0-20 m to 20-100 m and to the observed phytoplankton net growth during spring (data see https://doi.org/10.6084/m9.figshare.11830464.v1).

Appendix 2: Trait data

Tab. S1: Morphotype number and name, its assigned trait values of defense δ, maximum growth rate r (d^{-1}), phosphate affinity ($d^{-1}\mu mol^{-1}L$) and cell volume (μm^3) according to Bruggeman(10) and the taxonomic group of all 36 dominant phytoplankton morphotypes in Lake Constance. Bruggeman established a statistical model based on measured trait data and known phylogenetic relationships and trait correlations. We throughout used the values of the statistical model for consistency, although measurements for a few taxa deviated from them. Two morphotypes (*Navicula* spp. and *Cymbella ventricosa* & *C. prostrata*) were not listed by Bruggeman(10). Hence, we used trait values of the nearest genus for them (*Nitzschia* ssp. for both) having a similar longest linear dimension, cell volume and colony formation.

Morphotype number	Morphotype name	Defense	r	Phosphate affinity	Cell volume	Taxonomic group
1	*Anabaena* spp.	0.66	0.88	94	170	cyanobacteria
2	*Asterionella formosa*	0.79	1.6	56	810	diatoms
3	*Aulacoseira* spp.	0.77	1.5	18	870	diatoms
4	*Ceratium hirundinella*	0.89	0.24	1600	40,000	dinophytes
5	*Chlamydomonas* spp.	0.38	1.8	170	210	chlorophyta
6	*Chlorella* spp	0.69	1.7	45	28	chlorophyta
7	*Chrysochromulina parva*	0.73	1.0	160	250	haptophytes
8	*Cosmarium* spp.	0.84	0.95	72	5,700	chlorophyta
9	*Cryptomonas marssonii*	0.49	1.1	190	1,800	cryptomonads
10	*Cryptomonas rostratiformis*	0.5	1.1	200	3,200	cryptomonads
11	*Cryptomonas* spp.	0.45	1.2	140	1,700	cryptomonads
12	*Cyclotella* spp.	0.77	1.6	3.6	420	diatoms
13	*Cymbella ventricosa* & *C. prostrata*	0.54	1.8	150	320	diatoms
14	*Diatoma* spp.	0.76	1.4	210	1,400	diatoms
15	*Dinobryon* spp.	0.85	0.74	110	360	chrysophytes
16	*Erkenia subaequiciliata*	0.47	1.7	220	100	chrysophytes
17	*Eudorina elegans*	0.78	1.1	32	1,300	chlorophyta
18	*Fragilaria crotonensis*	0.84	1.3	50	1,200	diatoms
19	*Mallomonas* spp.	0.8	0.5	340	2,700	chrysophytes
20	*Mougeotia* spp.	0.77	1.5	87	1,200	chlorophyta
21	*Navicula* spp.	0.54	1.8	150	320	diatoms

22	*Nitzschia* spp.	0.54	1.8	150	320	diatoms
23	*Oocystis* spp.	0.6	1.5	190	510	chlorophyta
24	*Oscillatoria* spp.	0.53	1.0	99	23	cyanobacteria
25	*Pandorina morum*	0.82	0.92	46	2,300	chlorophyta
26	*Pediastrum* spp.	0.69	1.3	310	790	chlorophyta
27	*Peridinium* spp.	0.912	0.24	130	15,000	dinophytes
28	*Phacotus* spp.	0.34	1.6	430	400	chlorophyta
29	*Rhodomonas* spp.	0.1	1.7	550	250	cryptomonads
30	*Scenedesmus* spp.	0.63	2.1	45	160	chlorophyta
31	*Sphaerocystis schroeteri*	0.83	1.1	57	720	chlorophyta
32	*Staurastrum* spp.	0.85	0.84	150	12,000	chlorophyta
33	*Stephanodiscus neoastraea*	0.66	1.7	22	1,500	diatoms
34	*Stephanodiscus* spp.	0.64	1.8	13	580	diatoms
35	*Synedra* spp.	0.66	1.7	420	1,300	diatoms
36	*Tabellaria fenestrata*	0.8	1.2	210	2,700	diatoms

The concave trade-off between δ and r can be also found when including all trait data from Bruggeman(10) (Fig. S1a) or when the standard errors for the 36 morphotyptes are included (Fig. S1b).

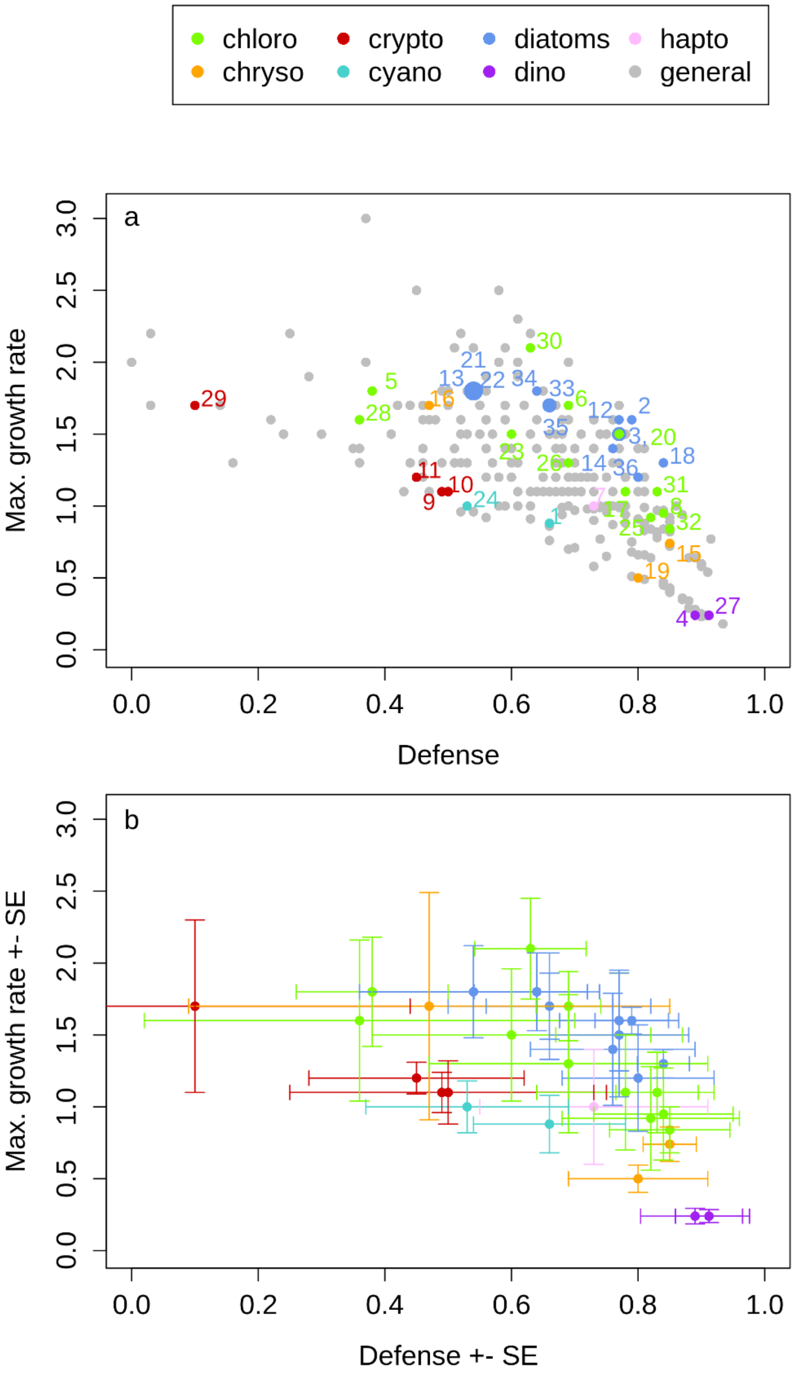

Fig. S1: Defense δ and maximum growth rate r (d^{-1}) of the 36 most abundant phytoplankton morphotypes in Lake Constance (colored dots). Colors indicate different taxonomic groups, i.e., chlorophyta, cryptomonads, chrysophytes, haptophytes, cyanobacteria, diatoms and dinophytes. (a) Including all other phytoplankton morphotypes/species available in Bruggeman(10) (grey dots). The numbers refer to the morphotype names listed in Tab. S1. Larger dots indicate that two or more morphotypes share the same trait combination. (b) The bars indicate the standard error of the derived trait values of the 36 morphotypes according to Bruggeman(10).

Appendix 3: Supporting results

We describe supporting results here, (1) correlations between phosphate affinity P and defence δ resp. maximum growth rate r, (2) the relationship of all three traits to cell volume, (3) the seasonal biomass-trait distribution considering all seven phases, the influence of re-oligotrophication on (4) the seasonal dynamics of r and δ, and (5) on the seasonal dynamics of P.

Correlations of defense and maximum growth rate with phosphate affinity

A concave trade-off curve was most obvious for the trade-off between δ and r (Fig. 3). In the trait space of δ and P, a similar pattern might be seen albeit with more scatter and one exception, *Ceratium hirundinella* being very defended and highly phosphate affine (Fig. S2a, $\rho = -0.28$, $p = 0.09$, Spearman rank correlation coefficient never biomass-weighted). The pattern for P and r is even more scattered (Fig. S2b, $\rho = -0.12$, $p = 0.5$).

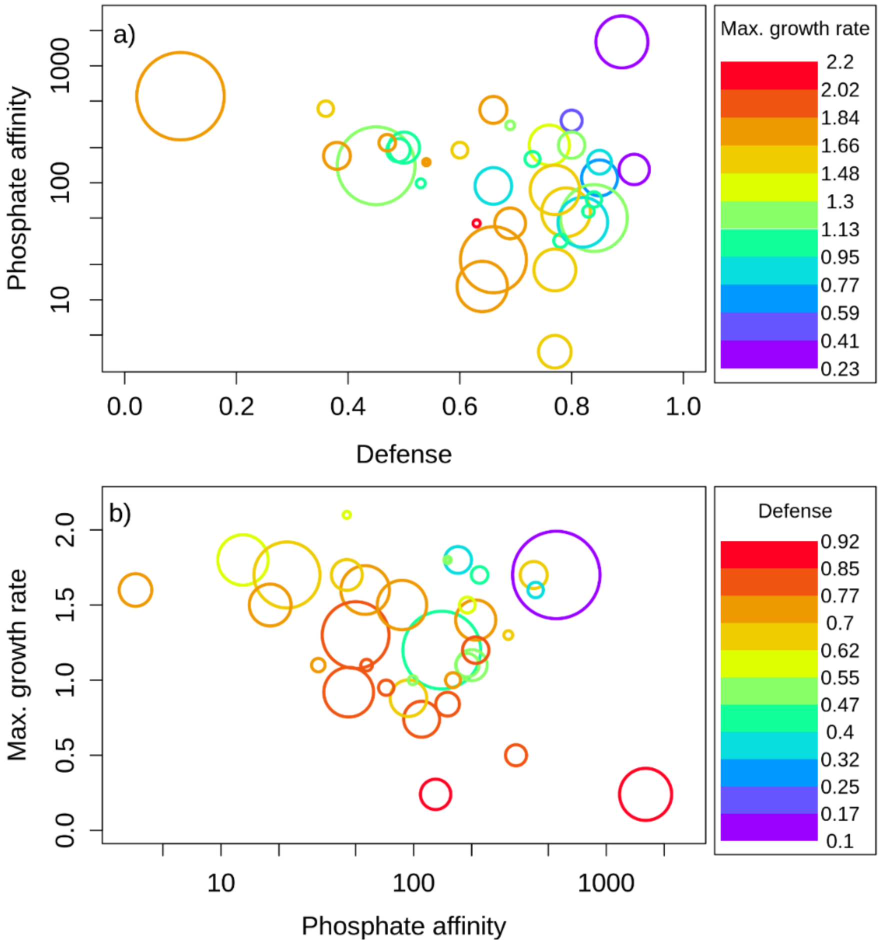

Fig. S2: Trait space of the 36 most abundant phytoplankton morphotypes in Lake Constance. Colors indicate third trait dimension. The area of the circles is scaled by the mean annual relative biomass of the

morphotypes. (a) Defense δ and phosphate affinity ($d^{-1}\mu mol^{-1}L$), color represents maximum growth rate r (d^{-1}). (b) Phosphate affinity ($d^{-1}\mu mol^{-1}L$) and maximum growth rate r (d^{-1}), color represents defense δ.

Trait correlations with cell volume

The correlation between each trait and cell volume as a master trait was tested (Fig. S3). Maximum growth rate r was negatively correlated to cell volume (ρ = -0.59, p = 10^{-4}), defense was positively correlated to cell volume (ρ = 0.49, p = 10^{-3}), while we found no correlation for phosphate affinity (ρ = 0.22, p = 0.19). Thus, the trade-off between δ and r may partially arise from the weak correlations of both traits with cell size. However, the large scatter in the relationship between δ and cell size (Fig. S3b) shows that other defense mechanism are important as well, e.g. cell shape, formation of filaments. Hence we used trait data instead of the approximation cell size.

Furthermore, cell size as a functional trait is harder to link directly to a certain environmental driver being sensitive to multiple factors as a ‘master trait’, compared to, e.g. defense being selected by high grazing pressure, or phosphate affinity being selected by phosphate depletion. Therefore, we do not include cell size in our main consideration of how the community trait composition responds to seasonal environmental changes, but we use cell size more as a ‘master trait’ providing to some extent a potential mechanism for the observed trade-off between defense and maximum growth rate.

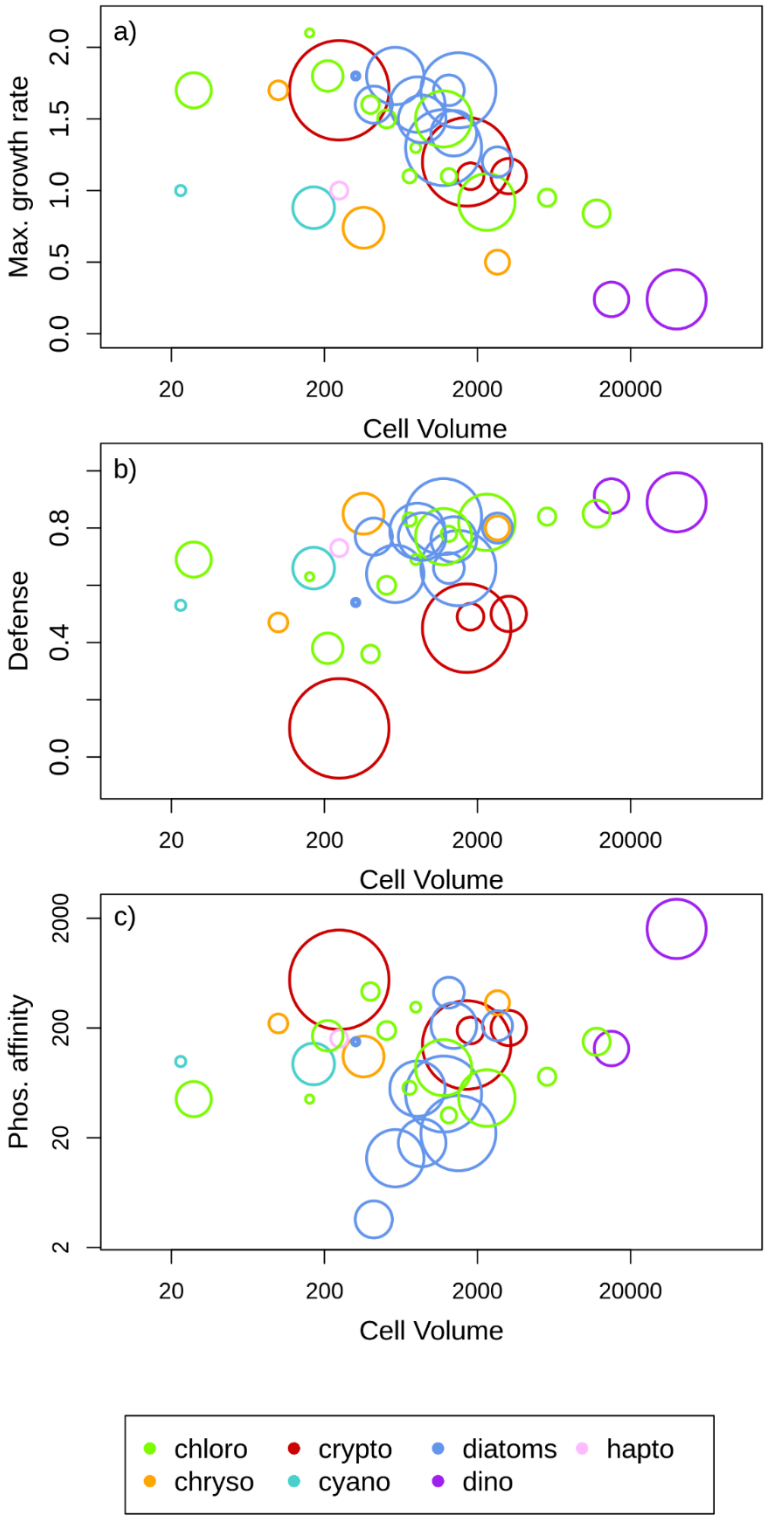

Fig. S3: Relationship between cell volume (µm³) and a) maximum growth rate r (d^{-1}), b) defense δ and c) phosphate affinity ($d^{-1}\mu mol^{-1}L$) for the 36 most abundant phytoplankton morphotypes in Lake Constance (colored circles). Colors indicate different taxonomic groups, i.e., chlorophyta, chrysophytes, cryptomonads, cyanobacteria, diatoms, dinophytes, and haptophytes and the area of the circles scales with mean annual relative biomasses.

Biomass-trait distributions for all seasonal phases

The biomass distribution in the δ-r trait space responded to the seasonally changing environment in a remarkably gradual and consistent way (Fig. 2, Fig. S4). In late winter and early spring, vertical mixing and the resulting high export of phytoplankton from the euphotic zone to deep water layers was a dominant driver of the phytoplankton community in deep Lake Constance while grazing pressure and nutrient depletion were very low. Morphotypes with high r being able to compensate for high losses and to exploit the high nutrient concentrations dominated, whereas morphotypes with low r and high δ were almost absent (Fig. S4a, b). This is reflected in the community average trait values (late winter: $\bar{\delta}$ = 0.51, $\bar{r}$ = 1.56 d^{-1}; early spring: $\bar{\delta}$ = 0.52, $\bar{r}$ = 1.57 d^{-1}). Morphotypes with low or high phosphate affinities had high biomasses, indicating the absence of a selection pressure on this trait. During late spring, grazing pressure increased mostly by ciliates (Fig. 2) but did not initiate a shift of the overall biomass distribution towards higher δ ($\bar{\delta}$ = 0.48, $\bar{r}$ = 1.55 d^{-1}) (Fig. S4c). During the clear-water phase (CWP), the grazing pressure was at its annual maximum (Fig. 2). The community average maximum growth rate decreased slightly ($\bar{r}$ = 1.35 d^{-1}) while the mean defense level did not change ($\bar{\delta}$ = 0.48) despite the high grazing pressure (Fig. S4d), probably due to a delayed numerical response of highly defended but slowly growing morphotypes. In summer, nutrient depletion and grazing pressure were the dominant drivers of phytoplankton (Fig. 2). The biomass shifted towards morphotypes with intermediate or high δ and accordingly low r (Fig S4e, $\bar{\delta}$ = 0.69, $\bar{r}$ = 1.18 d^{-1}). Morphotypes with intermediate to high phosphate affinities gained in importance in line with nutrient depletion. In autumn, nutrient depletion and grazing were still mainly driving the phytoplankton community but declined compared to summer (Fig. 2). This resulted in a slight increase of morphotypes with lower δ and higher r ($\bar{\delta}$ = 0.62, $\bar{r}$ = 1.28 d^{-1}) (Fig. S4f). In early winter, vertical mixing again represented the most important driver and nutrient concentrations were high. Morphotypes with high r, intermediate δ and no clear signal in phosphate affinity contributed again a high share to the total phytoplankton biomass (Fig. S4g, $\bar{\delta}$ = 0.56, $\bar{r}$ = 1.40 d^{-1}).

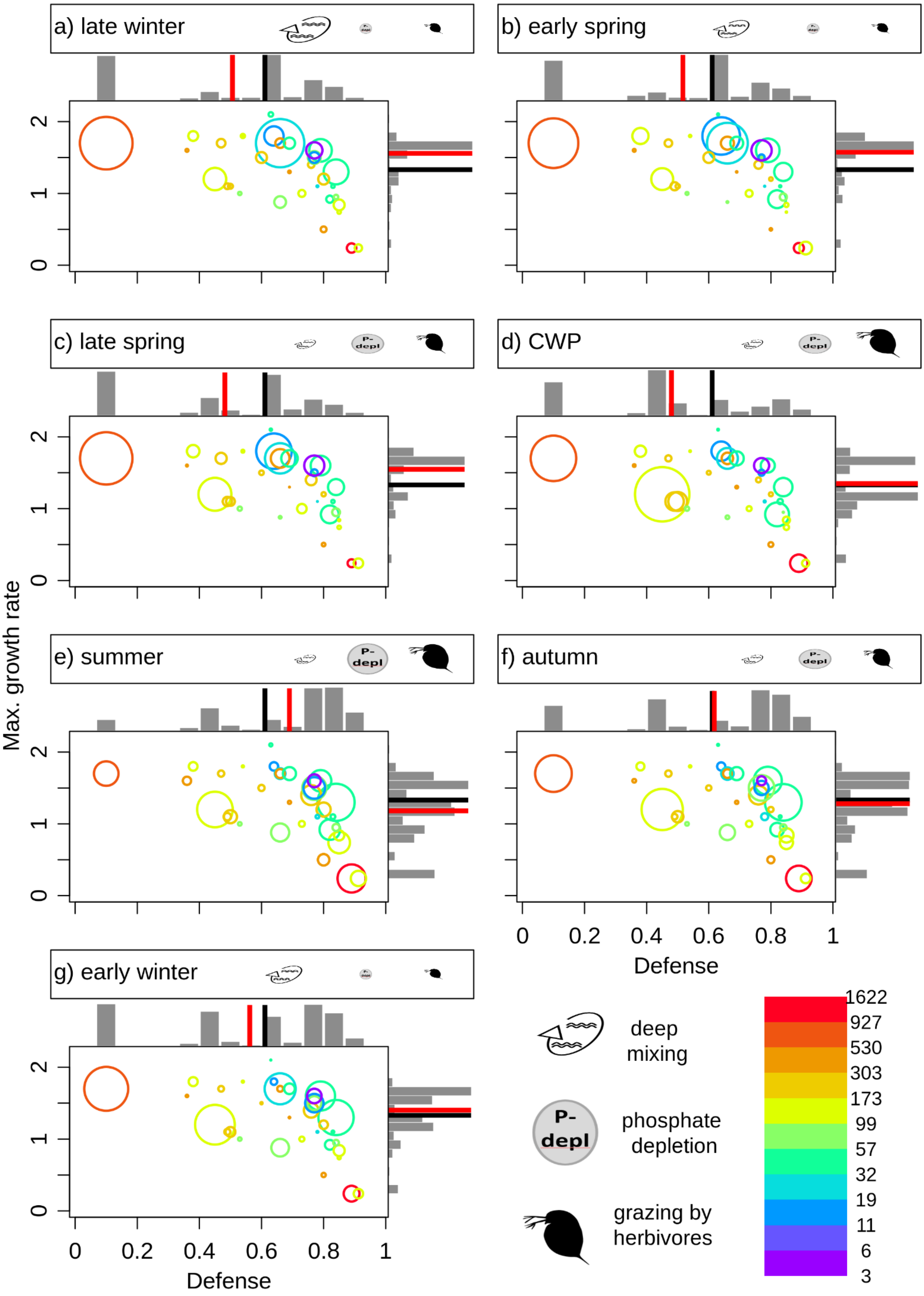

a) late winter
b) early spring
c) late spring
d) CWP
e) summer
f) autumn
g) early winter
P-depl
Max. growth rate
Defense
0
0.2
0.4
0.6
0.8
1
2
deep mixing
phosphate depletion
grazing by herbivores
1622
927
530
303
173
99
57
32
19
11
6
3

Fig. S4: Positions of the 36 most abundant phytoplankton morphotypes in Lake Constance in the trait space of defense δ and maximum growth rate r (d^{-1}) for seven seasonal phases. Colors indicate the morphotypes' phosphate affinity and the area of the circles the mean relative biomasses of each phase. The bars display the relative biomass distribution along the two trait axes in each phase. The red lines in the bar plots mark the phase means of the community average trait values and the black lines display the annual means of the community average trait values as a reference ($\bar{\delta}$ = 0.61, $\bar{r}$ = 1.33). The icons represent the dominant drivers of the phytoplankton community (vertical mixing, phosphate depletion, grazing by herbivores) and their size indicates their relative importance for phytoplankton net growth in each phase.

Influence of re-oligotrophication on seasonal dynamics of defense and maximum growth rate

Concurrent to the re-oligotrophication of the lake during the study period, the pattern of the seasonal biomass-trait distribution only marginally changed (Fig. S5). In early spring in 1979 – 1988, the morphotypes were on average slightly less defended than on average in 1989-1999 due to *Rhodomonas spp*. being more abundant, while intermediately defended diatoms were less common. These changes did not alter the average maximum growth rate. In summer in 1979-1988, the average maximum growth rate was higher than in 1989-1999, e.g. due to a higher share of the relative fast- growing *Fragilaria crotonensis*, whereas in 1989-1999 slow-growing *Ceratium hirundinella* was relatively more abundant. These changes did not affect the average level of defense. To conclude, the changes in the species composition observed during the re-oligotrophication did not change the overall seasonal pattern with a dominance of fast growing, undefended morphotypes in early spring and of slow growing, highly defended morphotypes in summer.

Our explanation for the lack of a clear responsiveness of the defence-growth rate trait distribution is that the grazing pressure likely changed little during most of the investigation period lasting from 1979-1999. We know that from 1987-1998 neither total ciliate biomass nor species composition changed significantly(11). The crustaceans, i.e. the other important group of herbivores, had lower abundances in 1997-1998 than in 1979-1996 during July to September. These fairly constant biomasses of herbivores fit with the measurements of 14C primary production from 1980-1996. During this time primary production declined (only) during summer by only 25%(12). We presume that potential effects on the trait distribution are too small to be clearly visible. On the other hand, the persistence of the trait distribution and its seasonal dynamics suggest that trophic interactions played a major role in this lake during the whole study period, as supported by numerous other studies(13).

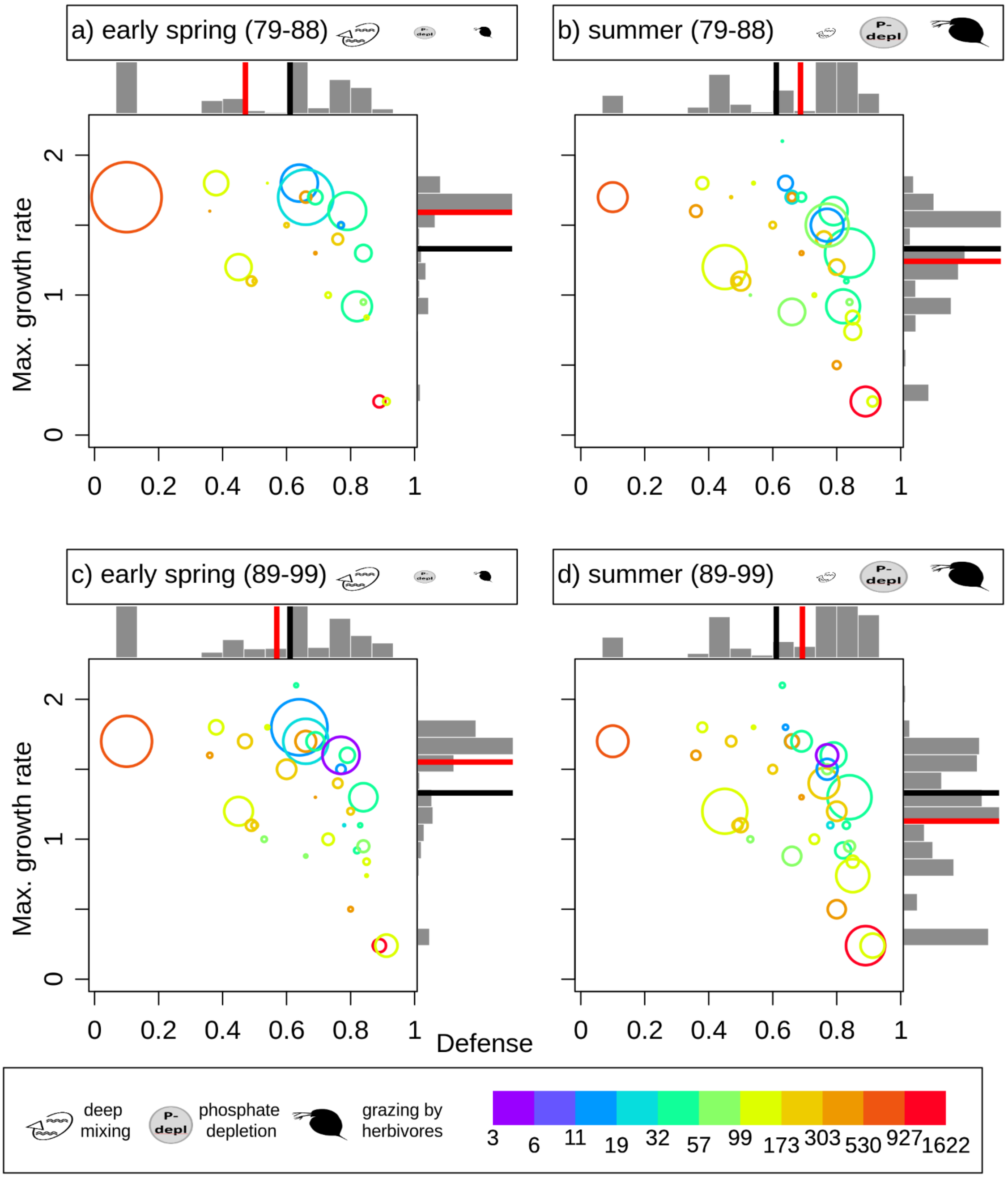

Fig. S5: Positions in the trait space of defense δ and maximum growth rate r (d^{-1}) and mean relative biomasses (scaling the area of the circles) of the 36 most abundant phytoplankton morphotypes in Lake Constance for two seasonal phases (early spring and summer) in 1979-1988 (a,b) and 1989-1999 (c,d). Colors indicate the morphotypes' phosphate affinity. The icons represent the dominant drivers of the phytoplankton community (vertical mixing, phosphate depletion, grazing by herbivores) and their size indicates their relative importance for phytoplankton net growth in each phase. The bars display the relative biomass distribution along the two trait axes in each phase. The red lines in the bar plots mark the phase mean of thecommunity average trait values and the black lines display the annual means of the community average trait values as a reference.

Influence of re-oligotrophication on seasonal dynamics of phosphate affinity

In summer, SRP dropped always below 2 µg P/l in the surface layer and we measured a strong increase in the cellular phytoplankton C:P ratio in summer 1995. Nevertheless we found no distinct seasonal pattern in community average phosphate affinity when considering all years together or the years 1979-1988 (Fig. S6a,b). For the years 1989-1999 we see a slight increase in phosphate affinity during the season, which results in somewhat higher values in summer and autumn compared to 1979-1988. The overall changes of the community average trait values are small relative to the entire trait range (3 -1600 $d^{-1}\mu mol^{-1}L$).

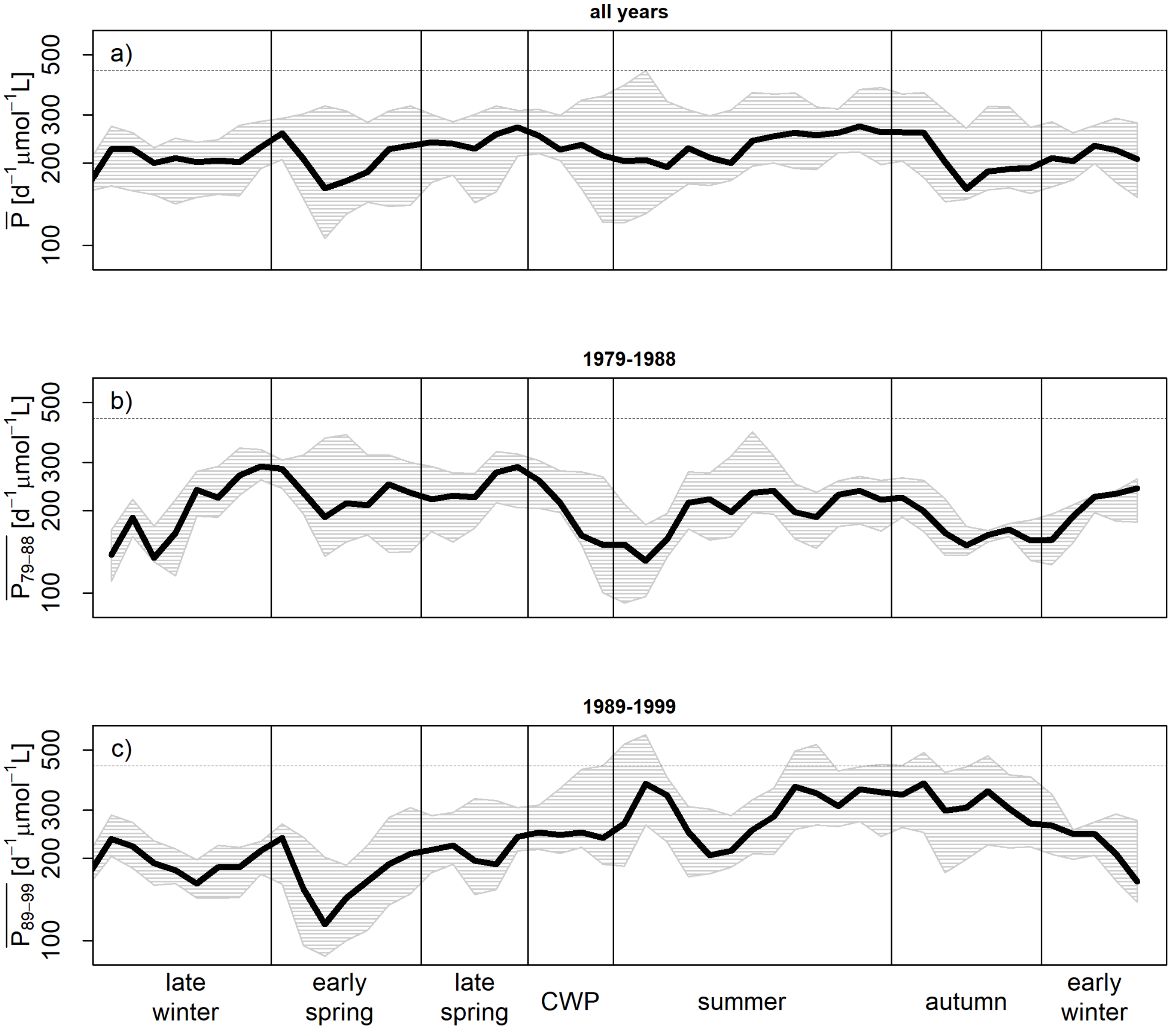

Fig. S6: Seasonal dynamics of the interannual median (black lines) and quartiles (shaded areas) of the phytoplankton community average phosphate affinity $\bar{P}$ in a standardized year of a) all years, b) 1979-1988 and c)1989-1999. CWP denotes the clear-water phase. The dashed line represents maximum of the upper quartile for all years.

Appendix 4: Model description

The model

The following model equations describe the biomass dynamics of N phytoplankton species P_i and one zooplankton group Z:

$$\frac{dP_i}{dt} = \left(r_i \frac{R}{K+R} - \frac{G\ (1-\delta_i)\ Z}{H+\sum_{i=1}^{N} P_i} - m_P\right) P_i \quad \text{(S1)}$$

$$\frac{dZ}{dt} = \left(\varepsilon\ \frac{G\ \sum_{i=1}^{N}[(1-\delta_i)P_i]}{H+\sum_{i=1}^{N} P_i} - m_z\right) Z$$

The phytoplankton growth is limited by nutrients, described by a Monod term. By assuming a fixed pool of nutrients, we can write the available (dissolved) nutrient concentration as $R = R_{max} - \sum_{i=1}^{N} P_i - \frac{1}{\varepsilon} Z$, that is, the total amount of nutrients R_{max} subtracted by the nutrients fixed in biomass of phytoplankton and zooplankton(14). The nutrient concentration is written in units of phytoplankton biomass, i.e. R_{max} represents the maximum phytoplankton biomass obtainable from the nutrient pool in the absence of mortality. The phytoplankton species differ in their maximum growth rates r_i, but share the same half-saturation constant for nutrient uptake K (in units of phytoplankton biomass, see above) and natural mortality m_P. Hence, the species with the highest r_i, performing well at high resource availability, is also the superior competitor under strong resource depletion (i.e. it has the lowest R^*) in the model. The grazing of zooplankton on phytoplankton is described by a Holling type II function with the maximum grazing rate G and the half-saturation constant H. Phytoplankton species have different values of defense δ_i against zooplankton. We assume that defended phytoplankton cells also demand handling time of the predator equal to that of undefended phytoplankton but without energy gain because unselective feeders, which dominate in Lake Constance, are probably not able to discriminate between them and attack both(15). Accordingly, δ_i gives the probability of not being consumed (i.e., not ingested or digested) and surviving when attacked with values ranging between 0 (undefended) and 1 (completely defended). The probability of being consumed is then given by $1 - \delta_i$, which scales the maximum grazing rate (see Eq. S1) and corresponds to the 'edibility', typically used in a limnetic context(10). The conversion efficiency of consumed phytoplankton into zooplankton biomass ε is assumed to be equal among the phytoplankton species. m_z represents the zooplankton mortality.

Trade-off curve

The trade-off curve between defense and maximum growth rate is given by the function

$r_i = b\,(0.9 - \delta_i)^a + c$ (S2)

where a denotes the shape parameter, b the slope parameter and c the maximum growth rate of the most defended species ($\delta_i = 0.9$). If $a < 1$, the trade-off curve is concave. $a > 1$ gives a convex trade-off curve and $a = 1$ a linear one. We assume a concave trade-off curve with $a = 0.2$, $b = 1.6\,d^{-1}$ and $c = 0.5\,d^{-1}$ approximately reflecting the trade-off curve found in the trait data (Fig. 3a). For comparison, we consider also a convex trade-off curve with $a = 2$, $b = 1.92\,d^{-1}$ and $c = 0.5\,d^{-1}$, that crosses the concave trade-off curve at minimal and maximal defense levels (i.e. shares the same endpoints).

Parametrization and initialization

We considered different phytoplankton species with trait values spanning the whole feasible trait space. We determined the species trait values according to the following procedure: First, we defined a 15x15 grid of trait combinations covering the whole trait space (δ_i between 0 and 0.9, r_i between 0.0 and 2.1 d^{-1}). Second, we extracted only the feasible trait combinations below the trade-off curve. Third, we added 15 trait combinations exactly on the trade-off curve, equally spaced along the whole defense axis, which resulted in a total number of 199 trait combinations representing different phytoplankton species ($N = 199$).

Based on measurements conducted at Lake Constance(13,16,17), we parametrized the model as follows: $R_{max} = 500\,mg\,C\,m^{-3}$, $K = 50\,mg\,C\,m^{-3}$, $G = 1.3\,d^{-1}$, $H = 80\,mg\,C\,m^{-3}$, $\varepsilon = 0.3$, $m_P = 0.2\,d^{-1}$, and $m_Z = 0.14\,d^{-1}$ (spring scenario, low grazing pressure) or $m_Z = 0.04\,d^{-1}$ (summer scenario, high grazing pressure). We initialized the model with random values from a uniform distribution between 0.1 and 4 $mg\,C\,m^{-3}$ for each P_i and between 1 and 20 $mg\,C\,m^{-3}$ for Z.

Numerical integration

The numerical integrations of the model were done with the ode45 solver of the deSolve package in R(18). We run the simulations for 10,000 days and calculated the mean biomasses of the last 1000 days to detect the phytoplankton species dominating in the long term. Furthermore, we checked which species survive in the short term, that is, within the first 100 days. The extinction threshold was set to $10^{-4}\,mg\,C\,m^{-3}$. We performed 50 simulations with different random initial conditions and averaged the mean biomasses and the time until extinction among all simulations.

Appendix 5: Phytoplankton biomass dynamics

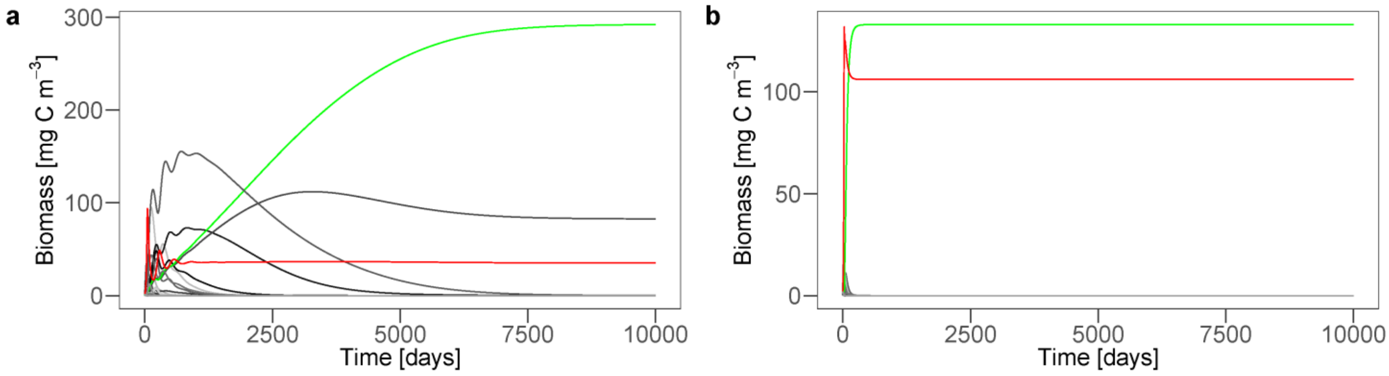

Fig. S7: Simulated long-term biomass dynamics for a concave trade-off curve under (a) low and (b) high grazing pressure (one sample of randomized initial conditions). Grey and green lines represent phytoplankton species with different trait combinations, where the green line marks the most dominant one. The red line corresponds to herbivorous zooplankton. (a) At low grazing pressure, two phytoplankton species with slightly different intermediate defense levels coexist. Their biomasses and the biomass of the herbivorous zooplankton reach a steady-state. (b) At high grazing pressure, only one species, with a higher defense level compared to the species coexisting in (a), survives in the phytoplankton. Its biomass and the biomass of the zooplankton are again in equilibrium.

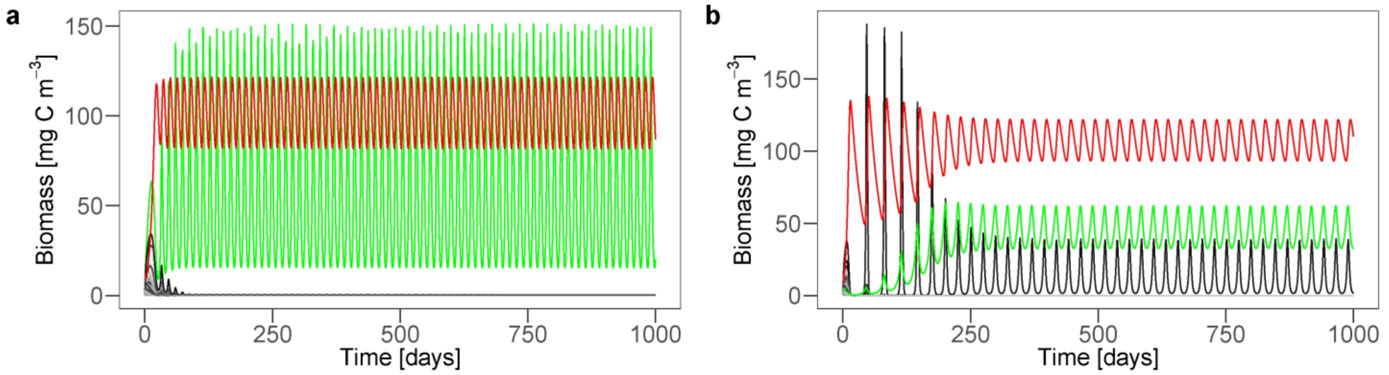

Fig. S8: Biomass dynamics in the first 1000 days of the simulation for a convex trade-off curve under (a) low and (b) high grazing pressure (one sample of randomized initial conditions). Grey and green lines represent phytoplankton species with different trait combinations, where the green line marks the most dominant one. The red line corresponds to herbivorous zooplankton. (a) At low grazing pressure, only a completely undefended phytoplankton species survives and the biomasses of that species and the zooplankton cycle. (b) At high grazing pressure, a completely undefended and a completely defended phytoplankton species (i.e. both extremes) coexist. Their biomasses and the biomass of the predator cycle.

References

1. Rocha MR, Vasseur DA, Gaedke U. Seasonal Variations Alter the Impact of Functional Traits on Plankton Dynamics. Swenson NG, editor. PLoS One. 2012 Dec 12;7(12):e51257.

2. Straile D. Biomass allocation and carbon flow in the pelagic food web of Lake Constance. Arch Hydrobiol Spec Issues Adv Limnol. 1998;53:545–63.

3. Boit A, Gaedke U. Benchmarking successional progress in a quantitative food web. PLoS One. 2014;9(2).

4. Gaedke U, Klauschies T. Analyzing the shape of observed trait distributions enables a data-based moment closure of aggregate models. Limnol Oceanogr Methods. 2017;15(12):979–94.

5. Weithoff G, Gaedke U. Mean functional traits of lake phytoplankton reflect seasonal and inter-annual changes in nutrients, climate and herbivory. J Plankton Res. 2017;39(3):509–17.

6. Weithoff G, Rocha MR, Gaedke U. Comparing seasonal dynamics of functional and taxonomic diversity reveals the driving forces underlying phytoplankton community structure. Freshw Biol. 2015 Apr;60(4):758–67.

7. Hochstädter S. Seasonal changes of C:P ratios of seston, bacteria, phytoplankton and zooplankton in a deep, mesotrophic lake. Freshw Biol. 2000 Jul;44(3):453–63.

8. Bäuerle E, Ollinger D, Ilmberger J. Some meteorological, hydrological and hydrodynamical aspects of Upper Lake Constance. Arch Hydrobiol Spec Issues Adv Limnol. 1998;53:31–83.

9. Gaedke U, Ollinger D, Straile D, Bäuerle E. The impact of weather conditions on the seasonal plankton development. Arch fuer Hydrobiol. 1998;53:565–85.

10. Bruggeman J. A phylogenetic approach to the estimation of phytoplankton traits. J Phycol. 2011 Feb;47(1):52–65.

11. Gaedke U, Wickham SA. Ciliate dynamics in response to changing biotic and abiotic conditions in a large, deep lake (Lake Constance). Aquat Microb Biol. 2004;34:247–61.

12. Häse C, Gaedke U, Seifried A, Beese B, Tilzer MM. Phytoplankton response to re-oligotrophication in large and deep Lake Constance: Photosynthetic rates and chlorophyll concentrations. Arch Hydrobiol Spec Issues Adv Limnol. 1998;53:159–78.

13. Gaedke U, Hochstädter S, Straile D. Interplay between energy limitation and nutritional deficiency: Empirical data and food web models. Ecol Monogr. 2002;72(2):251–70.

14. Klauschies T, Gaedke U. Nutrient retention by predators undermines predator coexistence on one prey. Theor Ecol. 2019 Oct 19;535195.

15. Ehrlich E, Gaedke U. Not attackable or not crackable-How pre- and post-attack defenses with different competition costs affect prey coexistence and population dynamics. Ecol Evol. 2018

Jul;8(13):6625–37.

16. Gaedke U, Straile D. Seasonal changes of trophic transfer efficiencies in a plankton food web derived from biomass size distributions and network analysis. Ecol Modell. 1994 Sep;75–76:435–45.

17. Gaedke U. Functional and taxonomical properties of the phytoplankton community: Interannual variability and response to re-oligotrophication. Arch fuer Hydrobiol. 1998;53:119–41.

18. Soetaert K, Petzoldt T, Setzer RW. Package deSolve: Solving Initial Value Differential Equations in R. J Stat Softw. 2010;33(9):1–25.
